# Small RNA sequencing-microarray analyses in Parkinson leukocytes reveal deep brain stimulation-induced splicing changes that classify brain region transcriptomes

**DOI:** 10.3389/fnmol.2013.00010

**Published:** 2013-05-13

**Authors:** Lilach Soreq, Nathan Salomonis, Michal Bronstein, David S. Greenberg, Zvi Israel, Hagai Bergman, Hermona Soreq

**Affiliations:** ^1^Department of Medical Neurobiology, Hadassah Faculty of Medicine, The Hebrew University of JerusalemJerusalem, Israel; ^2^Division of Genomic Medicine, The Gladstone Institute of Cardiovascular DiseaseSan Francisco, CA, USA; ^3^The Center for Genomic Technologies, The Institute of Life Sciences, The Hebrew University of JerusalemJerusalem, Israel; ^4^Department of Biological Chemistry, The Life Sciences Institute, The Hebrew University of JerusalemJerusalem, Israel; ^5^Department of Neurosurgery, The Centre for Functional and Restorative Neurosurgery, Hadassah University HospitalJerusalem, Israel; ^6^The Edmond and Lili Safra Center for Brain Sciences, The Hebrew University of JerusalemJerusalem, Israel

**Keywords:** deep brain stimulation, high throughput sequencing, leukocytes, miRNAs, Parkinson's disease, splice junction microarrays, substantia nigra

## Abstract

MicroRNAs (miRNAs) are key post transcriptional regulators of their multiple target genes. However, the detailed profile of miRNA expression in Parkinson's disease, the second most common neurodegenerative disease worldwide and the first motor disorder has not been charted yet. Here, we report comprehensive miRNA profiling by next-generation small-RNA sequencing, combined with targets inspection by splice-junction and exon arrays interrogating leukocyte RNA in Parkinson's disease patients before and after deep brain stimulation (DBS) treatment and of matched healthy control volunteers (HC). RNA-Seq analysis identified 254 miRNAs and 79 passenger strand forms as expressed in blood leukocytes, 16 of which were modified in patients pre-treatment as compared to HC. 11 miRNAs were modified following brain stimulation 5 of which were changed inversely to the disease induced changes. Stimulation cessation further induced changes in 11 miRNAs. Transcript isoform abundance analysis yielded 332 changed isoforms in patients compared to HC, which classified brain transcriptomes of 47 PD and control independent microarrays. Functional enrichment analysis highlighted mitochondrion organization. DBS induced 155 splice changes, enriched in ubiquitin homeostasis. Cellular composition analysis revealed immune cell activity pre and post treatment. Overall, 217 disease and 74 treatment alternative isoforms were predictably targeted by modified miRNAs within both 3′ and 5′ untranslated ends and coding sequence sites. The stimulation-induced network sustained 4 miRNAs and 7 transcripts of the disease network. We believe that the presented dynamic networks provide a novel avenue for identifying disease and treatment-related therapeutic targets. Furthermore, the identification of these networks is a major step forward in the road for understanding the molecular basis for neurological and neurodegenerative diseases and assessment of the impact of brain stimulation on human diseases.

## Introduction

Parkinson's disease (PD) is the second most prevalent neurodegenerative disease worldwide and the first movement disorder. PD is mainly expressed in progressive, deliberating motor symptoms, initiating with tremor at rest, slowness of movement and rigidity which develops into flexed posture, loss of postural reflexes and a freezing phenomenon (Fahn and Elton, 1987). Notably, the cardinal motor symptoms appear only years after initiation of the disease process. As the disease presents other central signs such as depression, sleep disorders (Bernal-Pacheco et al., 2012) and dementia (Goldman and Litvan, 2011) as well as peripheral symptoms (e.g., digestive and olfactory impairments) (Braak and Del Tredici, 2008), it is becoming increasingly clear that PD is not a pure motor disorder. In fact, it may initiate many years prior to the appearance of the motor symptoms (Hawkes et al., 2010). However, current methods do not allow early or accurate diagnosis and moreover, the genes and mechanisms that lead to the massive dopaminergic neuronal loss in the damaged PD brain are still largely obscure. The growing prevalence of PD worldwide introduces a strong incentive to find both disease biomarkers and future therapeutic targets.

MicroRNAs (miRNAs) are key post transcriptional regulators. Since their discovery 20 years ago (Lee et al., 1993), it has been well demonstrated that they play essential roles in development (Reinhart et al., 2000) as well as in human diseases, for example in cancer (Mraz et al., 2009; Li et al., 2013; Pu et al., 2013), as well as Alzheimer's disease and other central nervous system disorders (Maes et al., 2009). A growing body of evidence now suggests that peripheral miRNA changes may co-occur with the onset of PD (Doxakis, 2010; Martins et al., 2011). MiRNAs mediate post-transcriptional regulation of protein-coding genes by primarily binding to short sequence motifs found typically in the 3′ un-translated region of target mRNAs. Depending on the degree of sequence complementation, this leads to translational inhibition, mRNA destabilization or degradation. Since their discovery, miRNAs have emerged as crucial regulators of gene expression, and a single miRNA can concurrently regulate hundreds of target mRNAs. However, despite their biologic importance, determining global miRNA profiles and functional miRNA-target interactions remained a major challenge. Correspondingly, so far only about 20% of the known human miRNAs were found as constituting biological networks of functionally-associated molecules (Satoh and Tabunoki, 2011).

PD-related miRNAs have been characterized in midbrain dopaminergic neurons (Kim et al., 2007; Minones-Moyano et al., 2011), and were found to post-transcriptionally regulate the key PD genes LRRK2 (Gehrke et al., 2010) and SNCA (Doxakis, 2010). Also, down-regulation of miR-34b/c was found to modulate mitochondrial function in PD (Martins et al., 2011) and deletion of hsa-let-7 caused toxic effects similar to those of pathogenic LRRK2 (Gehrke et al., 2010) in a Drosophila PD model. However, post-mortem brain tissue is not readily accessible, and entails RNA quality concerns (Atz et al., 2007). Blood leukocytes, however, are readily obtained, and the RNA can be obtained at high quality from them. Recently, miRNAs were characterized in whole blood of PD patients (Martins et al., 2011) by microarrays and quantitative real time PCR (Margis and Rieder, 2011). RNA-Seq provides an unbiased, high resolution, accurate means for profiling miRNAs (Pritchard et al., 2012) and thus for investigating the miRNA mediated pathogenesis of complex diseases such as PD. Purported advantages of small RNA sequencing for this purpose include generation of data unlimited by genome annotations or probe affinities. Analyses of genome-wide small RNA transcriptome provide an accurate and comprehensive approach of globally characterizing miRNA expression in disease under different conditions. Yet, the global profile of miRNAs was not characterized yet by comprehensive RNA high throughput sequencing of PD blood cells.

Alternative splicing (AS) dramatically increases the complexity of the human transcriptome. Recent studies identified possible interactions between miRNAs and spliced targets (Salomonis et al., 2010; Irimia and Blencowe, 2012). Yet, while gene-expression changes have been characterized in PD blood cells using conventional microarrays (Scherzer et al., 2007; Soreq et al., 2008), such studies are blinded to alternative isoform regulation, which has been associated with at least 95% of the human genes (Pan et al., 2008). Deep brain stimulation (DBS) treatment dramatically improves the debilitating PD motor symptoms which are the cardinal disease symptoms (Bergman et al., 1990; Kingwell, 2011). Recently, we found disease-related transcription changes in PD leukocytes and demonstrated that DBS neurosurgery largely reversed the observed transcriptional changes (Soreq et al., 2012b). We have identified that a large number of predicted miRNA binding sites located in alternative exon regions.

Here, we performed a comparative miRNA, splicing and gene expression analysis of PD patients' blood leukocytes pre- and post-DBS with the electrical stimulus being on and following 1 h of stimulation cessation using high throughput RNA sequencing in conjunction with exon and splice junction microarrays of the same cells. Along with these samples, mRNA samples from matched healthy control volunteers were analysed.

We found significant differences in miRNA expression levels in PD, partially reversed by DBS, which co-occurred with high confidence changes in gene isoform expression levels and cell lineage composition. Our findings provide new insights for the molecular mechanisms underlying peripheral reactions to neurological and neurodegenerative diseases and opening new avenues for future development of both new diagnostic and therapeutic tools. We provide these data sets and analyses as a resource for understanding miRNA expression and splicing in Parkinson's patient's leukocytes pre- and post-treatment.

## Materials and methods

This study was authorized and approved by the Ethics committee of the human review board (Hadassah University Hospital, Ein-Kerem, approval number 07.09.07-6) in accordance with the Declaration of Helsinki principles. Following oral agreement all participants signed informed consent prior to inclusion in the study.

### Patient recruitment, DBS neurosurgery and clinical evaluation

Overall, seven PD male patients nominated for bilateral STN-DBS neurosurgery and six healthy age-matched male controls healthy control volunteers (HC) were recruited to the study. Clinical description appears under (Soreq et al., 2012b). Briefly, volunteers were assessed for their clinical background and state and fulfilled detailed medical history questionnaires. Patients with other medical conditions were excluded, including depression and past and current DSM Axis I and II psychological disorders (SM), chronic inflammatory disease, coagulation irregularities, previous malignancies or cardiac events, or any surgical procedure up to one year pre-DBS. All patients went through bilateral STN-DBS electrode implantation (Medtronics, USA) and were under dopamine replacement therapy (DRT) both pre- and post-DBS (on significantly reduced dosage post-DBS both on and off stimulation), the last medication administered at least 5 h pre-sampling. Patients further exhibited similar total white and red blood cell counts pre- and post-DBS. Blood samples were collected from each patient at three time points: (1) one day pre-DBS upon hospitalization, with medication; (2) post-DBS (range 6–18 weeks), when reaching optimal clinical state as evaluated by a neurologist and on a lower DRT dose, ON stimulus and (3) OFF stimulus, following 1 h while being OFF electrical stimulation (counted from stage 2). The clinical severity of disease symptoms was assessed by a neurologist for Unified PD Rating Scale (UPDRS-III) (Fahn and Elton, 1987) section 3 (motor section) in the tested stated (pre-DBS, post-DBS on and off stimulation). Controls were recruited among Hadassah hospital staff and researchers at the Edmond J. Safra Campus (Jerusalem). Exclusion criteria for the HC volunteers included smoking, chronic inflammatory diseases, drug or alcohol use, major depression, previous cardiac events and past year hospitalizations. The only group of other control volunteers that was included in this study is of healthy control volunteers, as samples from “sham” operated healthy (or patient) volunteers cannot be obtained or authorized by ethics committee. However, in addition to the healthy controls, we tested the leukocyte RNA from PD patients post-DBS also following 1 h of electrical stimulation cessation, to enable discrimination of the electrical stimulation effect from the effect of the presence of the stimulator in the brain *per-se*.

### Blood sample collection and RNA extraction

Blood collection was conducted between 10AM–14PM. Collected venous blood (9 ml blood using 4.5 ml EDTA (anti-coagulant tubes) was immediately filtered using the LeukoLock fractionation and stabilization kit™ (Ambion, Applied Biosystems, Inc., Foster City, CA) and incubated in RNALater (Ambion). Stabilized filters and serum samples were stored at −80°C. RNA extraction followed the manufacturers' alternative protocol instructions. Briefly, cells were flushed (TRI-Reagent™, Ambion) into 1-bromo-3-chloropropane-(BCP) containing 15 ml tubes and centrifuged. 0.5 and 1.25 volume water and ethanol were added to the aqueous phase. Samples were filtered through spin cartridges, stored in pre-heated 150 μl EDTA; RNA was quantified and examined on Bioanalyzer 2100 (Agilent, Santa Clara, CA, USA) and frozen in −80°C.

### RNA-Seq SREK library preparation and sequencing

Libraries for next generation sequencing (NGS) were prepared from total leukocyte RNA with the manufacturers' SREK (Small RNA Expression Kit) protocol (Applied Biosystems, Foster City, CA, USA). A total of 12 libraries were prepared: from RNA of blood leukocytes from three healthy volunteers and three PD patients in three states (pre-DBS, post-DBS on stimulation and following 1 h of stimulation cessation). The DNA Libraries were tested using a DNA 1000 Lab Chip on Bioanalyzer 2100. Briefly, the total RNA samples were hybridized with Adaptor Mix A which is a set of oligonucleotides with a single-stranded degenerate sequence at one end and a defined sequence required for SOLiD sequencing at the other end. The Adaptor Mix A constrains the orientation of the RNA in the ligation reaction such that hybridization with it yields template for SOLiD sequencing from the 5′ end of the sense strand. After hybridization, the adaptors are ligated to the small RNA molecules using Ligation Enzyme Mix, which is a mixture of an RNA Ligase and other components. Ligation requires an RNA molecule with a 5′-monophosphate and a 3′-hydroxyl end; therefore, most small RNAs can participate in this reaction, and intact mRNA molecules with a 5′ cap structure are excluded. Next, the small RNA population with ligated adaptors of each sample was reverse transcribed, to generate cDNA libraries. Treatment with RNase H followed, to digest the RNA from RNA/cDNA duplexes and to reduce the concentration of unligated adaptors and adaptor by-products. The cDNA libraries were amplified using bar-coded primer sets and 15–18 cycles of PCR. The amplified cDNA libraries were cleaned up and size selected from gel—PCR products ~105–150 bp were isolated, corresponding to inserts derived from the small RNA population. These contained each 90 base pairs of primers and adapter sequences. The amplified cDNA libraries generated with the SOLiD Small RNA Expression Kit were thereafter ready for attachment to beads at the emulsion PCR step of the SOLiD sample preparation workflow. The slides were analyzed on a SOLiD system V3.5 (Applied Biosystems). Applied Biosystems SOLiD sequencer generated 50-base read sequences as.csfasta files and the corresponding quality control (.qual) and statistic (.stat) files.

### Mapping to miRBase and to human reference genome

A total of 166,746,207 sequencing reads were obtained. All the reads (50 bp long) were subjected to trimming of the tag end terminal base pairs to remove these 15 bases. The trimmed (35 bases) reads were subjected to further trimming of the P1 start adapter as well as the SOLiD miRNA reverse primer sequences using CLC genomics workbench V4.0 [CLC bio, Cambridge, MA (Sakharkar et al., 2004a)] through local Smith–Watermann alignment. Overall, 50.92% of the reads contained adapter sequence and/or the SOLiD small RNA reverse primer. The 5′ terminal nucleotide was removed as well. This left 71,939,833 reads (average length of 21 bases). The minimum sampling count threshold was set to 1. The remaining reads obtained from each patient's library were first aligned against the human miRNA genome (miRBase release 15, which contained 940 Homo sapiens sequences). On average, 55% (range 44.9–61.3%) of the sequences of all libraries were mapped (Figure S1) and 50.94% (range 43.8–54.8%) of miRBase genes were detected (Figure S1). Of the mapped reads, on average 42.03% (range 39.6–45.9%) had perfect match to the aligned genes, 45.16% (range 42.5–55.3%)-one mismatch, 12.6% (range 0–18.6%)-2 mismatches and 0.18% (range 0.1–0.6%)-3 mismatches (Figure S1).

The full count information obtained from the alignment results is given under **Tables S4** and **S5**. Through the annotation and merge counts, only reads longer than 15 bases were analyzed. Match parameters included for mature length variants (IsomiRs)-additional 2 upstream and downstream bases, and 2 missing upstream and downstream bases. Alignment settings were not conducted on the color space. This allowed detection of mismatches up to the maximal allowed by the software (of 3 mismatches instead of 2). Annotated samples were grouped by both precursor and mature sequence identity.

Mapping to another repository of reference human genome database (Ambion, life technologies) which included 764 sequences of snoRNAs, tRNA, SINE/ALU sequences, LINEs and spliceosomal U genes (such as U1) yielded mapping to 293.3 (38.37%) filter database sequences on average across libraries. Only 151,136.8 (3.16%) reads of these that were not mapped to miRBase were mapped against this repository. Of them, 60.88% (range 42.2–68.9%) exhibited perfect match to the reference sequences, 30.94% (range 25.4–41.2%) with 1 mismatch, 6.24 (2.1–13.4%)-2 mismatches and 1.94% (0.5–3.3%)-3 mismatches (Figure S1). An average of 486 miRNAs per sample (range: 436–515) were detected in the analyzed libraries (Figures S2A–E: selected examples, full raw digital count quantification under **Tables S4** and **S5**).

### Differential expression analysis of next generation sequencing detected miRNAs

Overall, a total of 335 mature and 79 mature-star (mature 3′/5′ forms) that exhibited count of at least 1 per million reads in at least one sample were analyzed for differential expression between the different experimental conditions. Differential expression analysis of the miRNAs was conducted using the Bioconductor R package biocLite. The EdgeR (Robinson et al., 2010) and BaySeq (Hardcastle and Kelly, 2010) components were used for differential expression. 251 miRNAs were detected in all the samples, but the statistical methodologies applied allowed to analyze the detected miRNAs that presented count of at least 1 per million reads in at least one sample using adjacent statistical approach, to avoid false negative results. First, a combined data matrix of all the miRNAs detected in any of the samples was created. Then, the detected miRNAs were filtered for low count values such that only these with count ≥ 1 in at least 3 of the 12 libraries remained and 351 miRNAs were analyzed.

### Affymetrix HJAY splice junction microarray hybridizations and data pre-processing

The Affymetrix human junction arrays (HJAY) were obtained through collaboration with the EURASNET consortium and were used to assess genome-wide changes in exon and junction expression. Briefly, these microarrays interrogate 335,663 human transcripts from ~25,000 Ensembl genes, 260,488 junctions and 360,569 exons. Using the program AltAnalyze, probe set level RMA [17] expression and DAGB *p*-values were obtained by calling Affymetrix Power Tools (APT) [17]. From this probe set-level data, sample gene expression values were calculated from the mean intensity of constitutive annotated exon and junction probe sets in AltAnalyze.

### HJAY microarray profiling, database construction and analysis

HJAY profiling of blood leukocytes was conducted using exon array pre-prepared hybridization samples and Gene-Chip Whole Transcript Sense target Labeling assay kit (Affymetrix), as per manufacturers' instructions. The high-density HJAYs (Affymetrix) were washed and stained with streptavidin-phycoerythrin and signal amplification was done using a biotinylated anti-streptavidin antibody. The microarrays were scanned on an Affymetrix GeneChip Scanner 3000 7G scanner, according to the Affymetrix GeneChip Whole Transcript Sense Target Labeling Assay protocol for the GeneChip Exon 1.0 ST array (Lapuk et al., 2010). A total of 12 microarray samples were obtained. AltAnalyze probe set-to-exon associations were obtained by matching the annotated exon sequences (two exons for each junction probe sets) provided by Affymetrix to the reference Ensembl genome (version 62) for Affymetrix annotated gene symbols. In cases where probe sets aligned to an intron, a novel exon annotation indicating the relative intron position was assigned. Alternative exons, junctions, reciprocal probe sets and event-annotations (e.g., alternative cassette exon, alternative promoter) were obtained by comparing the exon-structure of mRNAs from Ensembl, the UCSC genome database and novel junctions assayed by the HJAY array, as previously described (Salomonis et al., 2010). Linear regression analysis was employed by an updated version of AltAnalyze to detect AS with a fold change of 2. *P* < 0.05 were considered significant. Additional methods details are provided at http://www.altanalyze.org/help_main.htm.

### Functional prediction analysis of the HJAY detected spliced transcripts

Each detected alternative event or alternative promoter selection (e.g., cassette exon, alternative 3′ end, alternative 5′ end, intron retention, bleeding exon, alternative C-terminal exon) or alternative promoter (alternative N-terminal exon or alternative promoter) of a pair of reciprocal junctions detected by the linear regression analysis was examined for putative protein domains or motifs and for miRNA binding sites. Alternative event annotations were obtained from both from Ensembl and UCSC Genome Browser databases as previously described for AltAnalyze version 1.0 (Salomonis et al., 2009).

### Functional enrichment analysis of the HJAY detected spliced transcripts

Functional *post-hoc* analysis of the alternative isoforms detected by linear regression analysis conducted for each comparison (e.g., patients pre-treatment as compared with controls) using Gene Ontology (GO) Elite (Zambon et al., 2012) was called directly from the adopted AltAnalyze version using Ensembl database. Genes cut-off parameters included minimal 2-fold change; and *t*-test raw *P*-value < 0.05 with minimum number of 3 changed genes defined. GO terms (Ashburner et al., 2000) and WikiPathways (Pico et al., 2008) were ranked by a combination of *z*-score (cut-off: 1.96) and gene number. Over-representation analysis (ORA) was conducted with 2000 permutations.

### Brain transcriptome microarray analysis

Raw expression data was obtained from the GEO dataset GSE8397 [citation: (Moran et al., 2006)], where Affymetrix HG_U133 array sets were used to determine the whole genome transcription profile of clinically documented and neuropathologically confirmed cases of sporadic Parkinson's disease as well as controls (and overall 47 samples). The .CEL files were normalized by sketch normalization and the expression of the 22,283 interrogated probe-sets (including 68 microarray internal control prone-sets) was quantified from 506,944 probes using the Probe Logarithmic Intensity Error estimation (PLIER) method (Affymetrix) while considering mismatch probes (Irizarry et al., 2003) (PLIER-MM).

### Exon microarrays hybridization and initial pre-processing

1 μg of total RNA was labeled using the Affymetrix exon array using whole transcripts sense targeting labeling assay according to the manufacturers' instructions; cDNA samples were hybridized to GeneChip® Exon_1.0_ST_Array (Affymetrix, Santa Clara, CA, USA) microarrays, and results were scanned (GeneChip scanner 30007G, 27 CEL files). Hybridizations were conducted in 7 batches. To prevent possible batch effect, each microarray hybridization included samples of patients from all experimental stages (pre, post-DBS on stimulation and post-DBS off stimulation) by random sampling. Quality assurance, normalization (quantile) and probe set summation (DABG iter-PLIER) were conducted using expression console (EC) 1.0 (Affymetrix, Santa Clara, CA). Only core level probe sets (*n* = 284.241) were included to summarize probe set level expression for 22,011 exon array transcript clusters (iter-PLIER, EC). Filtering out transcript clusters with no annotated gene symbols maintained 17,658 of 22,011 transcripts.

### Cellular lineage analysis of the exon microarrays data

To identify cellular composition, the new module of AltAnalyze, LineageProfiler, was used. To derive correlation scores to different cell types and tissues (lineages), a database reflecting the most specifically-expressed genes or exons present in each particular lineage, relative to all lineages types examined (ranging from 50 to 150), was created. The resulting database was a small subset of the original, containing the most informative markers. The exon microarrays RNA-profile expression data of patients in three states (pre-DBS, post-DBS on and following 1 h of electrical stimulation cessation-off stimulus) and HC volunteers was compared to the profile of each lineage only for these markers to derive correlation coefficients and resulting *Z* scores based on the distribution of values for each user RNA-profile. *Z* scores to each lineage were calculated from the distribution of Pearson correlation coefficients, specifically for each sample or condition analyzed. Lineage differences between conditions were specifically evaluated via the AltAnalyze GO-Elite module using the database of lineage specific markers for examined differentially expressed genes. The results were visualized as association scores at the level of hierarchically clustered cell types and curated lineage networks.

### miRNA: target predictions

Prediction of target gene sites for the miRNAs that exhibited differential expression in PD patients compared to control volunteers was conducted using the miRWalk repository which combines prediction data from 8 different prediction programs and adds inspection of all the gene regions including 5′ UTR and coding regions (Dweep et al., 2011). The analysis was conducted through a construction of a local MySQL database based on these predictions. For each miRNA that was detected as differentially expressed in the deep transcriptome of patients compared to control volunteers and post compared to pre-DBS, all the predicted targets were searched for and were filtered to these that were detected as alternatively spliced in the human junction microarrays for the corresponding tested condition. The final network of miRNA-spliced targets was analyzed and created through Cytoscape (Smoot et al., 2011).

### Accession codes

All the data discussed in this publication have been deposited in NCBI's Gene Expression Omnibus (Edgar et al., 2002):

1. High throughput SOLiD small RNA sequencing: http://www.ncbi.nlm.nih.gov/geo/query/acc.cgi?token=btahlkcwcuyyyvy&acc=GSE40915
2. Splice junction microarrays: http://www.ncbi.nlm.nih.gov/geo/query/acc.cgi?token=hhmvrgcsyyciexi&acc=GSE37591
3. Exon microarrays: http://www.ncbi.nlm.nih.gov/geo/query/acc.cgi?acc=GSE23676

## Results

### Differential expression of miRNAs in PD compared to healthy volunteers

**Figure 1:**
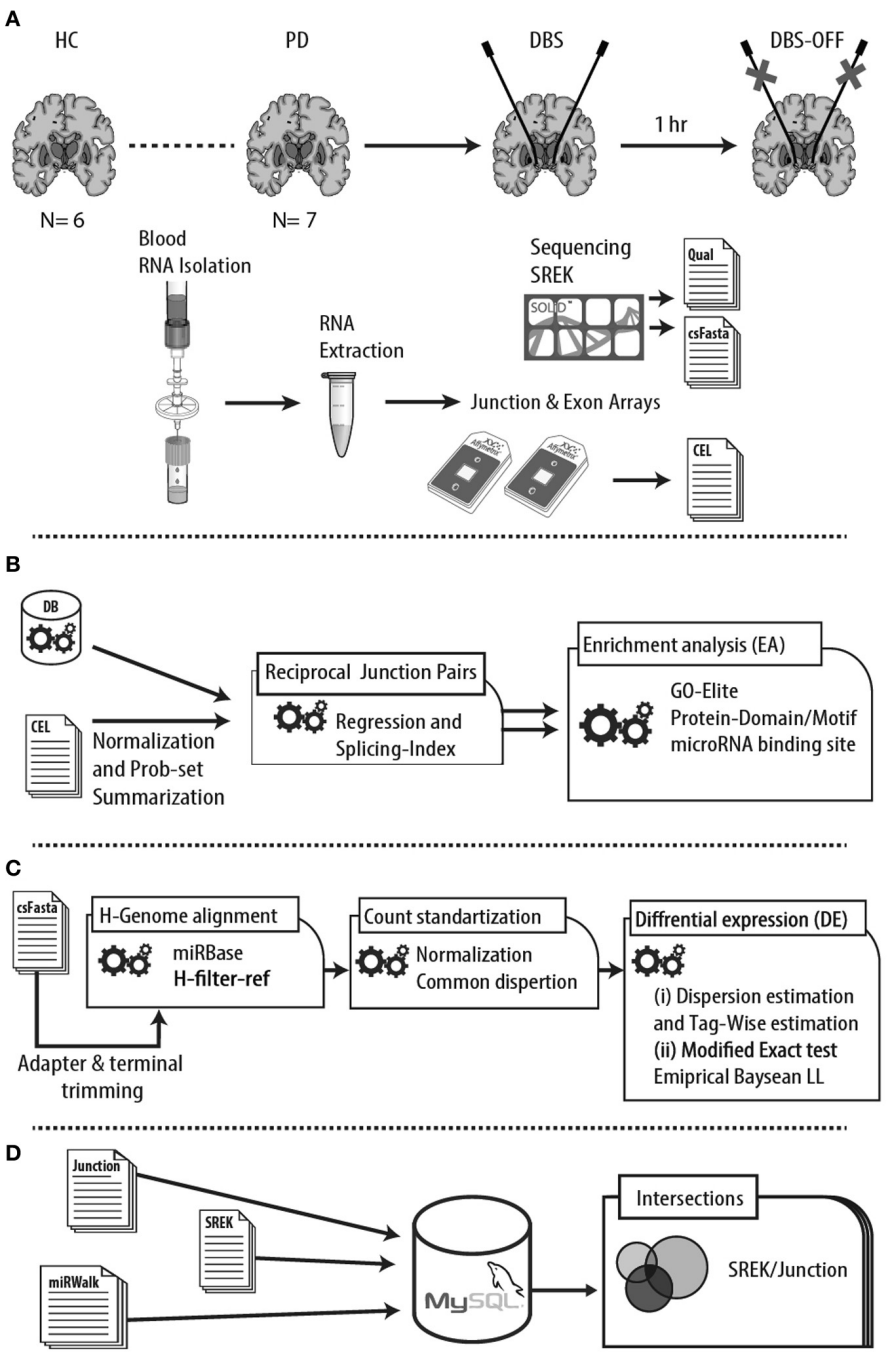
**Experimental design and analysis flow. (A)** RNA was prepared from filtered blood leukocytes isolated from whole blood samples of PD patients 1 day prior to DBS (PD), and post-DBS upon clinical stabilization, both on (DBS-ON) and following 1 h off electrical stimulation (DBS-OFF). Age- and gender-matched male healthy volunteers served as a control group (HC). Small RNA libraries were prepared using a Small RNA Expression Kit (SREK) and constructed according to the manufacturer's instructions. cDNA of patients pre- and post-DBS on stimulation and controls was also applied to Affymetrix human exon and junction prototype microarrays (HJAY). **(B)** The junction array datasets were processed in AltAnalyze using a previously described regression approach (Sugnet et al., 2006) on junction pairs, followed by functional enrichment analysis. Protein domains and enriched motifs and miRNA binding sites were also identified. **(C)** Reads obtained by high throughput sequencing were trimmed from adapter and end terminal bases and were aligned to miRBase and the human filter reference sequences database. The counts were standardized for common tag-wise dispersion and tested for differential expression using both dispersion estimation and Bayesian learning methods. **(D)** Predictions of miRNA: target interactions with the spliced transcripts were conducted by using the miRWalk algorithm which combines 8 established databases with predictions beyond the 3′ untranslated region (3′-UTR).

To delineate the expression of miRNAs in PD peripheral blood leukocytes, we employed SOLiD high throughput sequencing on small RNAs from peripheral blood leukocytes of PD patients followed by alignment to miRBase. The samples were obtained from three PD patients prior to DBS treatment, and upon symptoms stabilization following DBS neurosurgery, both on stimulation and following 1 hour off-electrical stimulation and as compared with matched healthy volunteers (clinical information under **Table S1**). The study design and overall analysis flow are given under Figure 1. The full mapping information and reads count are given under **Tables S2** and **S4**. Alignment to miRNABase detected 44.9–61.3% annotated reads (an average detection of 54.67% reads of each library) constituting 43.8–54.8% of miRBase (Figure S1 and **Table S2**). For comparison, we also aligned all the sequenced libraries against a reference database which contained 764 human filter sequences of other non-coding RNAs including snoRNAs, scRNAs, 5S_RNAs, tRNAs, SINE and ALU repeats, LINEs, spliceosomal U sequences (number 1,2,3,4,5,6,8,13,14, and 17) and snRNAs. These alignments yielded an average of 8.9% aligned reads of the total number of reads (with a minimum of 0.2% and a maximum of 3.16%) (**Table S3**). The raw read counts of all the detected miRNA and miRNA^*^ (also termed 3′/5′) mature forms (given under **Tables S4, S5** and Figures S2A–E) were filtered to include only those presenting at least 1 reads per million base pairs in at least three samples. Thus, 254 miRNAs, 2 recent miRNA-candidates which are highly likely tRNA fragments and 79 passenger strand miRNAs 3′/5′ forms (miRNA^*^) were analyzed (altogether 334). At the technical level, count data was discrete and skewed and was hence not well approximated by a normal distribution. To account for this, we applied a test based on the negative binomial distribution (a generalization of the Poisson model that recaptures biological variance correctly) (Robinson and Smyth, 2008). This test can effectively reflect the sample properties and detect differential expression. A linear modeling approach was applied for calculating fold changes and estimating standard errors, and was followed by an empirical Bayes smoothing (which provides more stable results for small sample sized data sets) to moderate the standard errors [16]. Also, sequenced libraries differ in the number of expressed transcripts. This fact could skew the differential expression calls due to over- and under-sampling (Robinson and Oshlack, 2010). To overcome this difficulty, we estimated tag-wise common dispersion to compute normalization factors for each library while accounting for library size. The common dispersion values enabled estimation of the bias using an empirical approach and were built into the library size. The computed concentrations were further subjected to an exact test to detect the top differentially expressed genes. This involved B statistic ranking (logarithm of the posterior odds that a gene is differentially expressed (Lonnstedt, 2002) through the EdgeR package (Robinson et al., 2010). Thus, the statistical approach applied here took into account both technical and biological variability of the digital count data.

**Figure 2:**
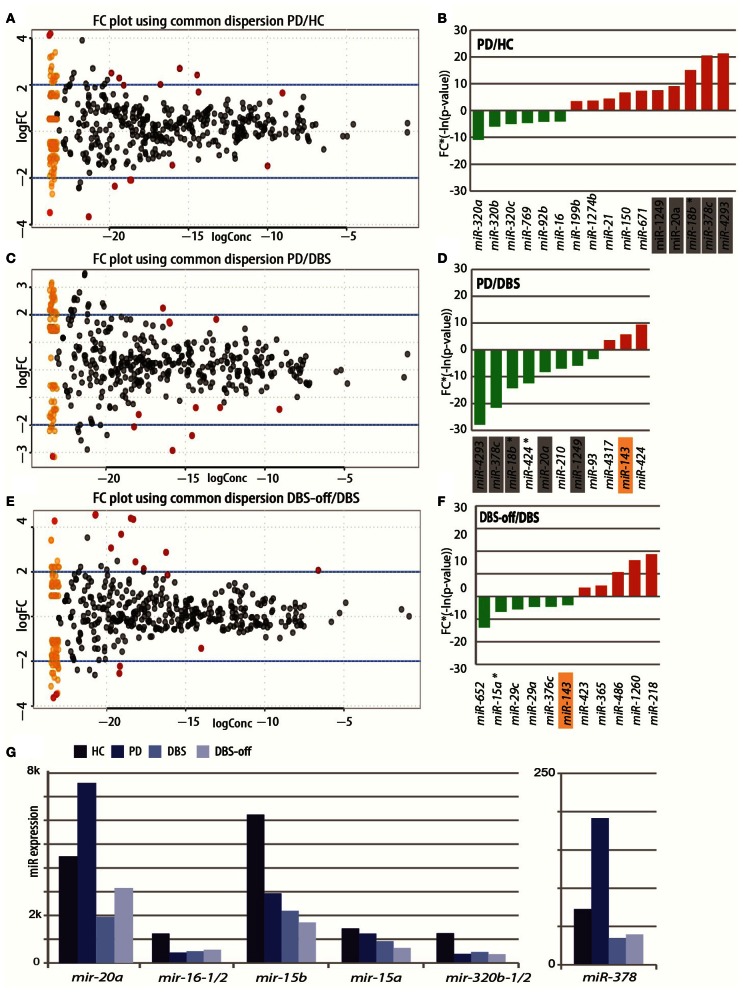
**Changed miRNA profiles in PD patients pre-and post-DBS and under stimulation cessation. (A,C,E)**. Log concentrations (*X*-axis) and log 2 fold change (FC) (*Y*-axis) of miRNAs detected as differentially expressed in **(A)** PD patient leukocytes pre- DBS as compared with healthy volunteers, **(C)** post-DBS on stimulation as compared to pre-DBS and **(E)** following 1 h off electrical stimulation. Red color denotes miRNAs that were detected as exhibiting a statistically significant change in each condition. The yellow dots represent all miRNAs that exhibited close to zero expression in one or both conditions (thus they cannot be plotted on a log scale). These condition-unique points in the plot are artificially represented by spreading them vertically as a “smear” on the left. **(B,D,F)** The fold change and *p*-value of miRNAs exhibiting down- or up-regulation in patients' pre-DBS compared to **(B)** healthy volunteers, **(D)** post-DBS on stimulation as compared to pre-DBS and **(E)** post-DBS off stimulation compared to on-stimulation **(F)**. The length of the bar denotes the statistical significance and the direction of change is denoted by positive or negative direction of the bar. *Y*-axis: -ln (*p*-value)*log fold change. Marked miRNA names: 5 (~30%) of the miRNAs that were detected as changed in PD patients were also detected as changed post-DBS and exhibited reverse direction of change compared to the disease state. **(G)** The non-normalized sequence read counts for selected changed miRNAs in the four tested states.

Taking a B-statistic threshold of one, 16 out of the identified 332 miRNAs and one of the tRNA fragments previously named hsa-mir-1280 (Schopman et al., 2010), were detected as differentially expressed between PD patients and healthy control volunteers (Figure 2A, full list under **Table S6**). Of the detected miRNAs, 12 were predicted to bind AS regions detected as changed in the junction arrays. Overall, 6 miRNAs were down regulated and 10 up regulated in PD patients (Figure 2B), some of which already shown as relevant to PD or other neurodegenerative diseases. For example, hsa-miR-16-1 and hsa-miR-16-2, two precursors that yield the same mature hsa-miR-16 that was recently identified as changed in PD blood cells (Margis and Rieder, 2011), were decreased in PD leukocytes (log2 fold change: −1.11, *p*-value: 0.026, expression under Figure 2B). Inversely, the neuro-inflammatory regulator hsa-miR-20a that is down-regulated in human aging (Hackl et al., 2010) and is affected in Alzheimer's disease (Delay et al., 2011) was up regulated in patients (log fold change: 1.5, *p*-value: 0.002). Elevated hsa-miR-20a-mediated suppression of the transcription factor E2F1 can also impair transcription (Brock et al., 2009), whereas potentiated targeting by mir-20a of the BMPR2 protein, a pathogenic hallmark of pulmonary hypertension may compete with BMPR2 regulation by the miR-17/92 cluster (Huang et al., 2012) which was also identified as changed in our PD patients data (log fold change: −1.16, *p*-value: 13.28). Among the changed miRNAs was also the miR-17/92 cluster, which is regulated by the pro-inflammatory cytokine *interleukin (Il)-6* (Brock et al., 2009). Intriguing, miR-320, which was modified in PD patients as compared with matched healthy control volunteers is included in a miRNA signature of prion-induced neurodegeneration (Saba et al., 2008), perhaps reflecting a feedback response aimed to avoid disease symptoms that was potentiated by DBS. Inversely, the tumorigenic miR-18b was elevated in PD (Guo et al., 2008). Additionally, the recently identified primate-specific hsa-miR-1274b (log fold change: 1.07, *p*-value: −11.79) which closely resembles a known tRNA was also up regulated, predicting global aberrations in miRNA metabolism. The PD-increased hsa-miR-21 (log fold change: 1.3, *p*-value: 0.02) also participates in a miRNA-based gene dys-regulation pathway in Huntington's disease (Johnson et al., 2008), and potentiated suppression by hsa-miR-21 and hsa-miR-150 of *PTEN* and *Huntingtin* would intercept T- and B- cells proliferation (Krichevsky and Gabriely, 2008; Lundstrom, 2011). Also, the reduced hsa-miR-378 (log fold change: 2.25, *p*-value: 0.000109) predicts impaired control over mitochondrial metabolism (Carrer et al., 2012) and negatively regulated NK cell cytotoxicity (Wang et al., 2012). Predicted decrease in another target, the Alzheimer's disease *APP* gene encoding for the amyloid precursor protein (Hébert et al., 2009) may indicate that at least some of these miRNA changes reflect a protective feedback response.

### DBS induces stimulus-dependent reversal of PD leukocyte miRNA changes

Patient leukocyte miRNAs were re-examined post DBS, upon symptoms stabilization. Similar analysis identified differential expression of 11 miRNAs, 8 down and 3 up-regulated as compared to the pre-DBS state (*p* < 0.05, B statistics, **Table S7** and Figure 2C) and the two tRNA fragments which were previously annotated as two new miRNAs (**Table S7**) (Schopman et al., 2010). Strikingly, 5 of the DBS-modified miRNAs were among those that were changed in PD compared to healthy volunteers but exhibited inverse direction of change post-DBS as compared with the PD-induced change (Figures 2C,D). For example, hsa-mir-20a and hsa-miR-378 increased in PD patients and decreased post-DBS, whereas the inflammatory-regulating hsa-miR-424 (Spinelli et al., 2013) showed no change in PD but it's passenger 3′ form (hsa-miR-18b-3p, previously called star form) exhibited down-regulation following DBS.

The DBS neurosurgery leads to greatly reduced medication dosage (which was also the case in our cohort), raising the possibility that the post-DBS changes were due to this reduced dose and/or to the surgical procedure itself. To challenge these questions, we also examined the small RNA transcriptome following 1-h stimulation cessation in the same group of patients. Disconnecting the electrical stimulation rapidly re-induces the motor disease symptoms that were apparent prior to the DBS treatment (such as resting tremor). Rapid and dynamic changes in miRNA expression were previously observed in several instances [e.g., (Perry et al., 2008; Pang et al., 2009)], but were never tested in live patients.

To isolate the effect of the electrical stimulation on the expression patterns of miRNAs in the blood we further analysed the small RNA transcriptome of patients' blood leukocytes following a 1 h of electrical stimulation cessation. Disconnecting the electrical stimulus notably induced in this short time lapse expression change in 11 miRNAs (six decreased and five increased) Figures 2E,F and **Table S8**). This indicated that the post-DBS miRNA changes were neither due to the lower medication dose nor to the surgical procedure, but rather reflected the retrieved motor symptoms under stimulus cessation. The modified leukocyte miRNAs also differed in their copy numbers (for example, hsa-miR-20a expressed higher as compared with hsa-miR-320, Figure 2G). One of the putative identified as changed miRNA (hsa-miR-1280) is a fragment of a tRNA (Griffiths-Jones et al., 2008). Disconnecting the electrical stimulus further reversed the DBS-mediated change in hsa-mir-143 (Figure 2F). Correspondingly, the post-DBS miRNA profile differed from that of healthy volunteers by only nine miRNAs (Figure S3) whereas 1 h OFF stimulation induced 4 miRNA count changes as compared with the healthy state including hsa-mir-320a and hsa-mir-320c (Figure S3). Taken together, these findings imply that miRNA profiles in both post-DBS and the OFF stimulus conditions differ from the pre-surgery disease state. The oncogenic hsa-mir-16 (Diniz et al., 2012), which was also observed in other PD blood cohort through RT-PCR as changed (Margis and Rieder, 2011) and the tumorigenic miR-18b (Guo et al., 2009).

### Splice junction microarray analysis identifies PD-related splicing changes, including in miRNA binding sites

**Figure 3:**
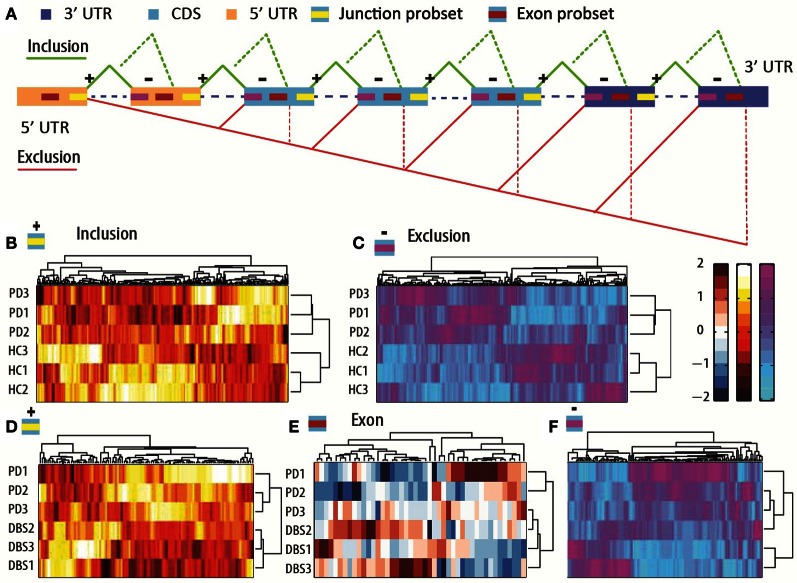
**Alternatively spliced exon-junction pairs classify PD and post-DBS samples correctly. (A)** A schematic illustration of the interrogated gene regions and possible combinations of inclusion and exclusion of junction and exon probe-sets. Dashed lines: possible inter-exonic Probe Selection Region (PSR) combinations with exclusion of junction probe-sets. A splice change is defined where bi-directional changes occur in the inclusion and the exclusion probe-sets (or alternatively, in a PSR and exclusion probe-sets) concurrently between two tested states. **(B)** The global expression of inclusion junction array probe sets that were detected as changed classified patients from healthy volunteers successfully. **(C)** Classification by the exclusion junction probe-sets. **(D)** Classification following DBS classified post-DBS from pre-treatment state by inclusion detected probe-sets. **(E)** Internal exon classification of the post-DBS from pre-DBS states by PSRs. **(F)** Classification by the DBS-modified exclusion junction probe-sets. Distance method for all the clusterograms: city block.

The observed miRNA leukocyte changes predicted corresponding changes in the levels of target transcripts of modified miRNAs. Given that the great majority of human genes are subject to splicing regulation, based on our exon microarray-based detection of exon microarrays-based detection of differential expression changes in PD leukocytes (Soreq et al., 2012c), we hypothesized that differential splicing regulation also exist in the patient's blood leukocytes. Furthermore, alternative isoforms that occur through alternative polyadenylation can also result in the removal or addition of microRNA binding sites in the 3′ UTR. Supporting this notion, the full splicing picture is far more complex than the resolution level of the exon arrays technology, since each splicing event may simultaneously cover one or more than one exon. To accurately identify the complex scope of inclusion and exclusion events in particular transcripts, we applied here human splice junction arrays (HJAY) (Sugnet et al., 2006), which simultaneously interrogate all of the exons and all of the splice junctions of each gene, enabling to construct a more robust combinatorial splicing picture (Figure 3A).

To detect alternative exons regulated from these arrays, the expression of pairs of opposing (reciprocal) exon-junctions were compared using a previously described linear regression approach (Sugnet et al., 2006) in the program AltAnalyze (Salomonis et al., 2009) for ~25,000 detected transcripts. This analysis revealed 478 disease-associated inclusion and exclusion events that occurred in 332 distinct genes, along with exons containing predicted miRNA binding sites (**Table S9**). These predictions correctly classified the patients from control groups by both the inclusion changed junction probe-sets (Figure 3B) and the exclusion ones (Figure 3C). Of these changes, 142 events were predicted to be functionally relevant (i.e., modifying a protein domain or binding site effects). 31 of those predicted change in a miRNA binding site. Statistical significance enrichment analysis identified nine miRNAs as frequently targeted at detected spliced transcripts: hsa-miR-769-3p (predicted to target four transcripts), hsa-miR-378 (with six targets) as well as hsa-mir-320, hsa-miR-92b-5p, hsa-miR-16, hsa-miR-150, hsa-miR-671, hsa-miR-20a, and hsa-miR-18b (The full list and adjusted *p*-values are given under **Table S10**). The enriched differentially expressed miRNAs were predicted to target 14 spliced transcripts, including the reactive oxygen species modulator *ROMO1*, the RNA-binding motif *RBM6* transcript involved in splicing (Heath et al., 2010) and the splicing factor *hnRNPA2B1*. Three newly annotated miRNAs were only identified in the small RNA sequencing data. PD-mediated inclusion of exon 12 in the exon 12 of the hnRNP gene *hnRNPA2B1* was detected by a mutual change in three junction probe-set pairs sharing the same second probe-set (**Table S9**). Notably, *hnRNPA2B1* has a role in regulating the biogenesis of miRNAs and in up-regulating hsa-miR-18a (Guil and Caceres, 2007) and down-regulating hsa-let-7 (Eddington et al., 2007). Recently, a link was found between mutation in this hnRNP and the neurological disease Amyotrophic lateral sclerosis (ALS) (Kim et al., 2013). Also, we recently found that hnRNPA2B1 is essential for neuronal wellbeing in Alzheimer's disease (Berson et al., 2012), compatible with the stress-associated changes in AS (Meshorer et al., 2002). The domain that we found here as included in PD patients extends the c-terminus of the hnRNPA2B1 protein that is necessary for nuclear localization and is sufficient for sending cytosolic proteins to the nucleus in a phosphorylation-regulated manner (Allemand et al., 2005). Furthermore, hnRNPA2B1 mediates mRNA shuttling to distal processes in both neuronal cells and oligodendrocytes (Han et al., 2010), and blockade of its function by splice-isoform-specific antibodies impaired the assembly of RNA granules. Notably, the extended domain spans putative binding sites for several PD-modified miRNAs (including hsa-miR-320a, hsa-miR-320b, and hsa-miR-320c), together indicating that the observed inclusion events may entail functional relevance.

*Post-hoc* functional GO analysis through the GO-Elite module of AltAnalyze of the regression detected genes revealed enrichment in transcripts of cellular components of the ubiquitin ligase complex (GO:0000151, *p* = 0.0165) and biological processes of mitochondrion organization (GO:0007005, *p* = 0.0185) and regulation of translation (GO:0006417, *p* = 0.015) (**Table S11**). The *FOXP1* transcript showed disease-mediated exclusion in PD patients, which was further annotated as replacing the N-terminus in the 679 amino acid long FOXP1 protein by an alternative N-terminus that yields a shorter protein product of 573 residues.

Independent exon-level splicing-index (Gardina et al., 2006) analysis of the exon probe sets present on the junction microarrays detected 202 PD-inducible AS events pre-DBS (**Table S12**). The detected exon interrogating probe-sets successfully classified patients from controls by expression level (Figure S4). The interrogated spliced genes that included the changed probe-sets exhibited enrichment in AS and apoptotic nuclear changes (**Table S13**). Of the exon level detected disease regulated genes, 34 were among these detected by the linear regression analysis of reciprocal junction pairs. However, only four of the detected alternative exons were annotated as overlapping with alternative polyadenylation sites, in the 3′ UTR (**Table S12**). Of the 202 identified events, 127 had functional predictions such as alternative terminals or non-sense-mediated decay regions (**Table S12**). Of these, 36 (~30%) were functionally predicted as miRNA binding sites (**Table S14**). Enrichment analysis detected 6 of the DBS-modified miRNAs as modified (having adjusted *p*-value < 0.05): hsa-miR-320 (a, b, and c) as predicted to bind 4 spliced transcripts including *hnRNPA2B1*, hsa-miR-378 (predicted to bind 6 spliced transcripts), hsa-miR-92b (predicted to bind 4 spliced transcripts), hsa-mir-150, hsa-miR-20a, and hsa-miR-18b (where hsa-miR-18b-3′ changed). Overall, the disease-modified miRNAs were predicted to target overall 11 spliced target transcripts, including *ROMO1* and *COL6A2* that were each predicted as targets of four different modified miRNAs (**Table S14**).

### Splice junction microarrays identify splice isoform changes following DBS including in miRNA predicted binding sites

To identify splicing changes induced by DBS, we compared the splice junctions of patients post-DBS to these of pre-DBS state. Linear regression analysis was conducted through AltAnalyze software package (Salomonis et al., 2010) which was adopted for the human junction arrays. The analysis demonstrated a more limited AS induction of 155 inclusion and exclusion events in 117 genes (**Table S15**). Of these, 140 entailed functional predictions, 109 changing protein domain(s) and 31 modifying miRNA binding sites (**Table S15**). For ~1/3rd of the detected splicing events, an additional internal exon probe set reproduced the junction-level results (45 out of 155), indicating higher confidence exon expression changes. Each of these measurement types (inclusion junction, exclusion junction, or inclusion exon) properly segregated the post-DBS samples from pre-DBS ones: the inclusion junction probe-sets (Figure 3D), the exon probe-sets (Figure 3E) and most distinctly, the exclusion probe-sets (Figure 3F), which showed an inverse pattern of change to that observed for PD, completely segregating the post-DBS samples from pre-DBS ones. The linear regression detected genes were enriched in the molecular processes of RNA import into nucleus and MAPK phosphatase export from nucleus as well as in ubiquitin homeostasis (**Table S16**).

DBS induced potentiated reduction in the junction pair measuring exclusion of the PD-included region of *hnRNPA2B1*, however, with two additionally decreased internal exons. Thus, PD *hnRNPA2B1* transcripts showed lower expression of the internal exon probe-set as compared with control volunteers, and post-DBS state caused a yet lower level of this internal region; and the opposite trend was found in the corresponding junction probes, reinforcing the conclusion that an exclusion event had occurred.

Exon level splicing-index analysis of PD patients post- compared to pre-DBS detected 131 AS events in 117 genes (**Table S17**), in 20 of them junctions were also detected as changed through the linear regression analysis of patients post- compared to pre-DBS. The detected exon-interrogating probe-sets successfully classified post- from pre-DBS samples (Figure S5). Intriguingly, 89 of the detected exons had functional transcript structure predictions. Two of these were annotated in Poly(A) sites (**Table S17**), and one of these predictions occured in the *Toll-like receptor (TLR)3*, a pro-inflammatory cytokine mediator (Butt et al., 2012). The NCBI web-based functional annotation tool DAVID (Database for Annotation, Visualization and Integrated Discovery) was used to investigate functional associations of splicing changes seen in the patients' blood leukocytes following DBS. Functional analysis using the DAVID EASE tool (Hosack et al., 2003) revealed that the DBS modified genes were functionally enriched in response to metal ion, AS, apoptosis, immune response and mitochondrion organization (**Table S18**). Enrichment analysis for miRNA binding sited in the detected genes detected 3 of the DBS-modified miRNAs: hsa-miR-20, hsa-mir-18, and hsa-miR-143 as highly predicted to bind AS targets detected by the exon level analysis (**Table S19**).

### PD brain region transcriptomes reveal disease-associated expression changes in the alternatively spliced leukocyte transcripts

**Figure 4:**
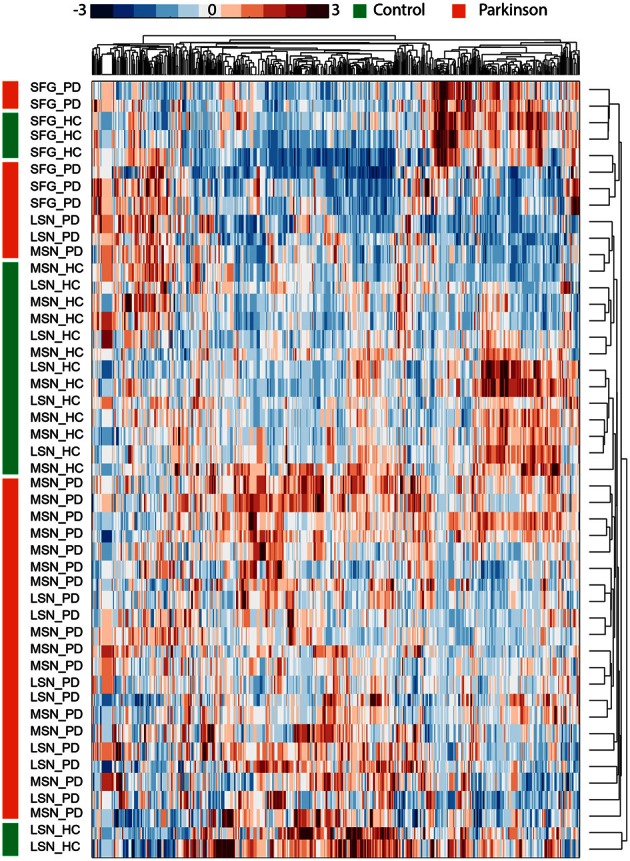
**Brain expression levels of spliced transcripts classify PD patients from control volunteers in a brain-region dependent manner.** Shown is a hierarchical classification in the noted brain regions of those same transcripts found to be alternatively spliced in PD leukocytes. Note that the hierarchical classifier successfully classified patients from healthy control volunteers based on expression levels obtained by 3′ microarrays interrogating three post mortem brain regions: superior frontal gyrus (SFG), medial substantia nigra (MSN) and lateral substantia nigra (LSN). Each row corresponds to one probe set interrogating a gene that was detected as alternatively spliced in leukocytes from patients and each column represents one sample of brain transcriptome from either patient (red) or healthy volunteer (green) from the independent cohort (Moran et al., 2006). The frontal brain region was correctly segregated from both substantia nigra regions by the expression data, and overall 43 of the 47 samples (~91.5%) were correctly classified as healthy or Parkinsonian ones.

Expression profiling of the alternatively spliced transcripts identified by junction microarray analyses of PD leukocytes was independently examined in a large microarray dataset of PD Patient's brain samples (Moran et al., 2006). The external dataset was composed of 3′ microarray data obtained from post mortem brain samples from PD patients and matched healthy control volunteers and included three brain regions: the lateral and medial substantia nigra and the superior frontal gyrus. The expression of 707 probe sets interrogating genes that were found as alternatively spliced (**Table S20**) successfully classified patients from control samples in this independent data-set (Figure 4), reflecting brain region specificity of the tested transcriptomes. The brain regions were further segregated by the blink classifier which separated the frontal gyrus from the substantia nigra (Figure 4). In the medial substantia nigra, all the patients were correctly classified apart from the control volunteers. Two lateral substantia nigra control samples were mis-classified with the patients, and two of eight patients were misclassified with the control volunteers based on the expression in the superior frontal gyrus. Thus, the vast majority of the samples were correctly classified as patients or control volunteers and overall over 91% accuracy of classification was obtained (Figure 4).

### Cellular composition of parkinson patients leukocytes

**Figure 5:**
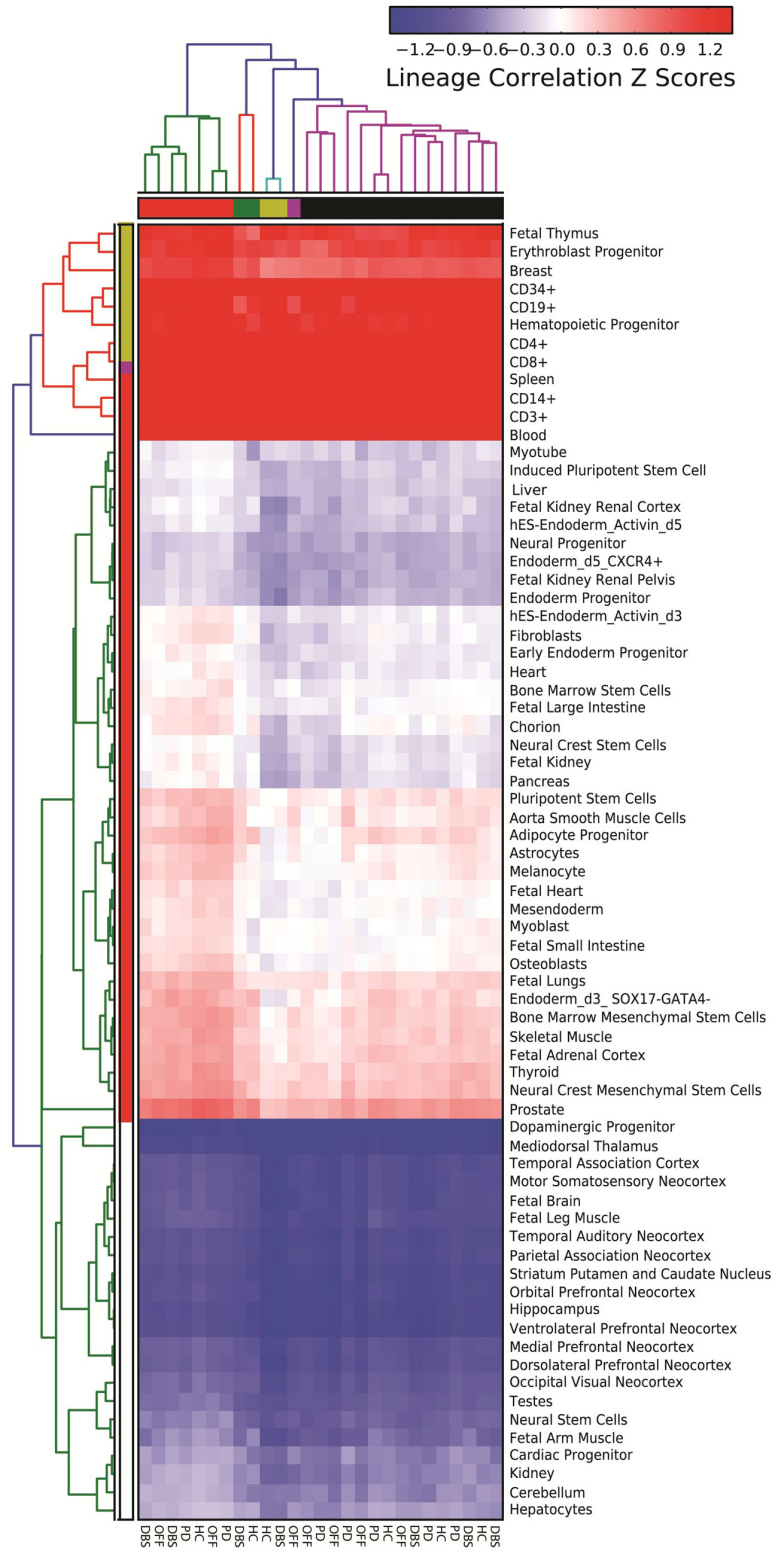
**Tissue and cell type origins of leukocyte-expressed AS-modified long transcripts.** Putative cell and tissue specific markers subjected to alternative splicing in the leukocyte RNA samples from tested patients and controls were hierarchically classified by exon array gene level expression (columns) and markers (rows). The color code scale presents lineage correlation *Z*-scores.

To identify the most likely cell types and tissues represented in the samples analysed by small RNA deep sequencing and microarrays, we have conducted a cell lineage analysis of the exon microarray data of seven patients examined in three states each and the six control volunteers using a newly developed module of AltAnalyze software package called LineageProfiler program. A large human and mouse compendium of publically available microarray datasets (from the GEO public repository) were used to identify gene sets for each cell type or tissue with the highest correlations to an idealized binary expression profile. The analysed samples included these tested also by junction microarrays and small RNA deep sequencing. The results were visualized as association scores at the level of hierarchically clustered cell types and curated lineage networks (Figure 5). The created database reflects the most specifically-expressed exons present in the samples, relative to all lineages types examined. We identified substantial immune cell activity in the patient's blood leukocytes both pre and post- treatment. Furthermore, most healthy individual samples were classified with post-treated patients and pre-treated patients with the off stimulation state based on the expression data of the marker genes.

### PD-spliced transcripts show multiple gene-wide binding sites for PD-modified miRNAs

**Figure 6:**
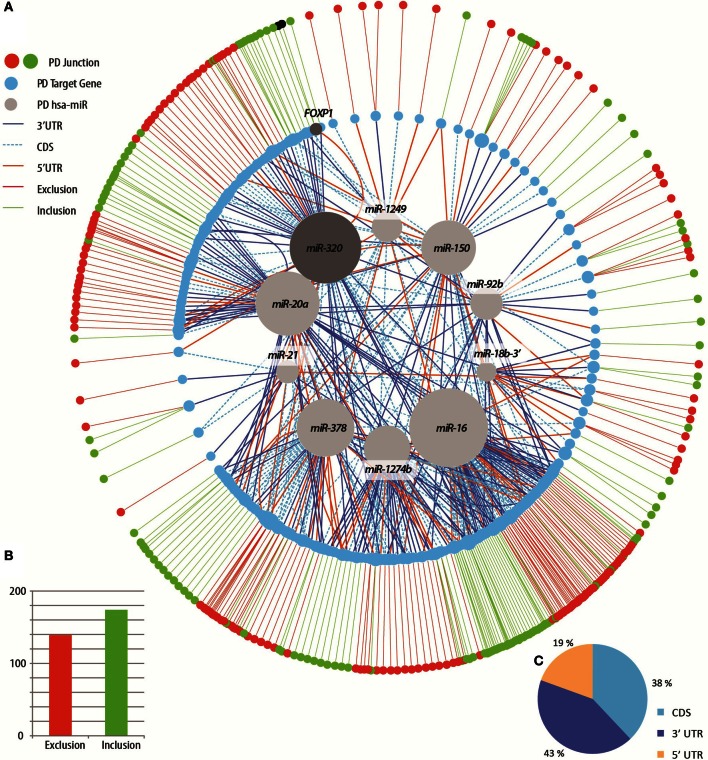
**A complex disease network of miRNA-spliced targets expanding beyond the 3′-UTR. (A)** Circular tree layout of the network of connections between the miRNAs detected as differentially expressed in PD patients to their predicted target genes that exhibited AS change in splice junction microarrays. External circle: red node color indicates exclusion event in the predicted target splice junction and green—exclusion. Larger node size indicates higher number of connections. The color of edges represents the gene region in which the binding is predicted to occur (5′, 3′-UTR or coding sequence, CDS). Middle circle: the predicted target genes. Gray circle color highlights miRNAs targeting the PD-related gene *FOXP1*, previously linked to miRNA regulation. Curved lines in the inner circle denote two different regions predicted to be bound (e.g., 3′ UTR and coding sequence). **(B)** PD patients exhibited more inclusion events (green) as compared with exclusion (red). **(C)** The types of events predicted to be regulated by the changed miRNAs: 3′ un-translated region (UTR) (dark blue), 5′ UTR (orange) or coding sequence (CDS, light blue).

Recent evidence indicates miRNA targeting beyond the 3′-UTR (Orom et al., 2008; Lee et al., 2009). Therefore, we extended the search for putatively changed miRNA-AS pairs to both the coding region and the 5′-UTR using miRWalk (Dweep et al., 2011). The miRWalk database, which predicts miRNA binding sites on the complete sequence of each gene, includes thousands of predicted targets for each of the PD-differentially expressed miRNAs. Using this information, we constructed a network of potential links between the 13 PD-differentially expressed miRNAs and the 217 target transcripts that showed AS changes in PD (Figure 6A), which exhibited more inclusion than exclusion events (Figure 6B). A total of 560 putative connections emerged (an average of 2.6 miRNA binding sites/target gene), with 43% of those on the 3′ UTR, 38% in the coding sequence and the remaining 19% in the 5′ UTR of the AS target genes (Figure 6C). This could not be explained by the relative length of the different gene regions of the identified links, since the average fraction (out of the total length for all genes) is 24% for the 3′ UTR, 69% for the coding sequence and only 7% for the 5′ UTR (Mazumder et al., 2003; Sakharkar et al., 2004b). The network showed a remarkable asymmetry containing the highest number of spliced junctions (68) in predicted targets for miR-16 and the lowest (1) for hsa-miR-18b-3p (previously called hsa-miR-18b^*^). Supporting the notion of disease relevance, this network included several known PD-related genes such as the transcriptional regulator *FOXP1* (Kim et al., 2007), that promotes midbrain dopaminergic identity in stem cells (Konstantoulas et al., 2010), which was identified as alternatively spliced in this study and was predicted as targeted by the PD dys-regulated hsa-miR-320 cluster. We conclude that at least some of the PD-modified miRNAs may potentially interact with part of the AS-modified transcripts in PD leukocytes.

### DBS-spliced transcripts show multiple gene-wide binding sites for DBS-modified miRNAs

**Figure 7:**
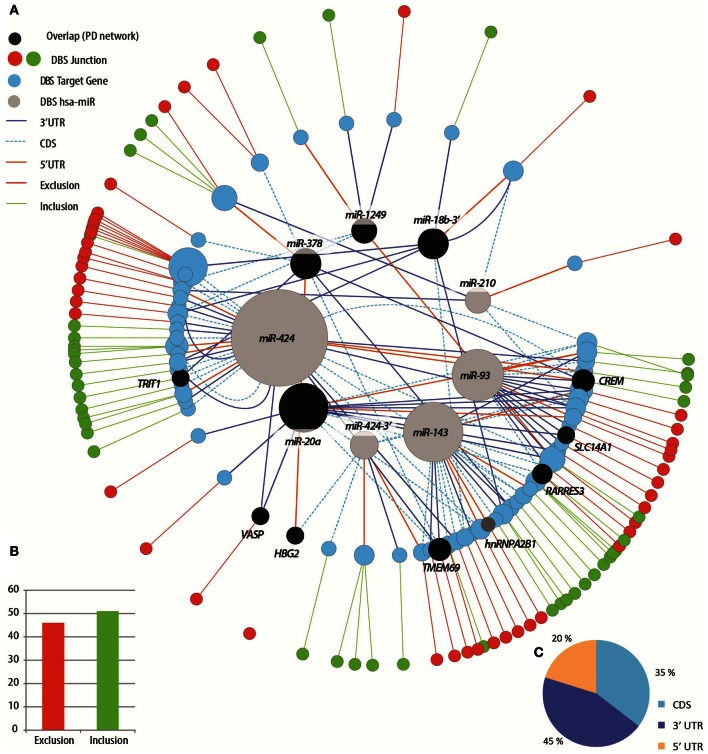
**The treatment induced network partially overlaps with the disease network. (A)** A circular tree layout network of connections between miRNAs that exhibited differential expression following DBS neurosurgery as compared with the pre-DBS (non-stimulated) state, and their predicted target genes that exhibited alternative splicing changes in PD patients following the DBS treatment. External circle: red node color indicates exclusion event in the predicted target splice junction and green—exclusion. Larger node size indicates higher number of connections. The color of edges represents the gene region in which the binding is predicted to occur (5′, 3′-UTR or coding sequence, CDS). Middle circle: the predicted target genes. Curved lines in the inner circle denote two different regions predicted to be bound (e.g., 3′ UTR and coding sequence). Black miRNAs and dark blue target genes: overlap with the patients compared to healthy network presented in Figure 6. **(B)** The number of exon exclusion (red) and exon inclusion (green) events detected in the predicted target genes that were detected by linear regression analysis of reciprocal junction pairs interrogated by junction microarrays (*Y*-axis: number of detected events). **(C)** The types of events predicted to be regulated by DBS modified miRNAs: 3′ un-translated region (UTR) (dark blue), 5′ UTR (orange) or coding sequence (CDS, light blue).

An independent network was constructed for the DBS-modified miRNAs and their DBS-spliced predicted targets (Figure 7A). Similar to the PD network, it included more AS predicted targets exhibiting inclusion (rather than exclusion) events (Figure 7B), with binding prediction again showing a decreasing order from the 3′ UTR (45%), coding sequence (35%) to the 5′ UTR (20%) (Figure 7C). The pair-sample DBS network was predictably thinner than the PD one as it avoided inter-individual variability and was based on smaller numbers of DBS-modified miRNAs and AS target transcripts. Nevertheless, this network as well showed asymmetry, with many more predicted AS targets for hsa-miR-424. The two networks shared four miRNAs that their predicted targets were modified both in PD and after DBS as compared with healthy volunteers. Of these, hsa-miR-378, and hsa-miR-20a were predicted to bind the same seven AS target genes, affecting the actin-associated protein VASP involved in axon guidance (Mohamed et al., 2012), the hemoglobin subunit gamma-2 *HBG2* gene, the retinoic acid receptor responder *RARRES3*, the androgen-regulated solute carrier *SLC14A1* (Vaarala et al., 2012), *TMEM69*, a dividing leukocytes biomarker (Solmi et al., 2006), the mitochondrial tRNA dimethyl-allyltransferase trit1 and the bZIP nuclear transcription factor. Other identified targets were the cAMP-responsive modulator *CREM*, which has known spliced isoform variants (Sanborn et al., 1997) and regulates target genes in different tissues (Fimia et al., 2000) and which associates with panic disorder (Domschke et al., 2003), depression and bipolar disorder (Crisafulli et al., 2012) and the schizophrenia-mutated GABA receptor modulator *DBI* which encodes a diazepam binding inhibitor (Niu et al., 2004).

We found three target genes (*HBG2, RARRES3*, and *VASP*) common to both networks to be regulated by the same miRNAs predicted to target the same junction probe-set pairs (Figure 7A), and in opposite directions from healthy controls to PD patients as compared with pre- to post-DBS. This is a remarkable overlap given that the DBS network was much narrower than the PD one and that the patient's pre-DBS changes were unpaired with the healthy volunteers.

The observed changes in miRNA-AS interactions in both PD and following DBS further raised the question if the predicted binding sites coincided with the modified splice junctions. Sequence comparisons of the miRNA seed regions with the junction probe sets that exhibited splicing changes identified such PD-and DBS-modified links in 34 (**Table S21**) and 33 events (**Table S22**) from 13 and 5 spliced transcripts, respectively. Given that this is only a small fraction of the modified transcripts in each case, this finding demonstrates that the great majority of the observed interactions did not involve active splicing events, while suggesting that direct miRNA-mediated splicing regulation is a viable, albeit exceptional case where the predicted binding site was located exactly in an alternatively spliced junction (**Tables S21** and **S22**). Exceptional PD-related examples of seemingly active miRNA-spliced transcripts predicted interactions include *USP13*, which promotes smooth (*SMO*) signaling by preventing its ubiquitination (Xia et al., 2012), *RGS3* which contributes to neural progenitor/stem cell regulation (Qiu et al., 2010) and *MGAT1*, involved in multiple sclerosis (Grigorian et al., 2012). The DBS-induced events occurred in the *NPC2* gene, which shares cholesterol interactions with *alpha-synuclein* (*SNCA*) (Liu et al., 2010), as well as in *HLX, C11orf5, CREM*, and the poly-A binding protein coding transcript *PABPC1L*.

## Discussion

Our study provides the first report of complete small RNA transcriptome sequencing of live human PD patients' blood leukocytes. Using a whole transcriptome high throughput sequencing technique in conjunction with analyses of both exon and junction microarrays interrogating RNA from the same cells of patients prior to brain stimulation treatment and post-treatment while being on and off electrical stimulation as well as of healthy volunteers, we characterized differential expression in miRNAs predicted to bind target transcripts that underwent splicing modifications. We have successfully classified microarray brain samples of an independent patients cohort based on the expression levels of genes that were found as alternatively spliced in blood leukocytes, at high accuracy of over 91%. Moreover, the dual comparative analysis of patients pre- and post- treatment as well as compared to healthy volunteers yielded insights into both disease and treatment mechanisms. Practically all of the human genes are simultaneously subject to post-transcriptional regulation by either AS or miRNAs thus both miRNAs and spliced isoforns represent promising new candidates for PD research.

For the small RNA sequencing, we used SOLiD genome analyser, obtaining ~106 reads per sample, which is considered a sufficient coverage for small RNA profiling, with above 50% average mapping to the miRBase database, detecting about half of miRBase miRNAs in the samples in up to millions of copies for few of them. For comparison, alignment to a different reference sequence database of other (non-miRNA) non-coding human RNAs yielded only an average of ~9% alignment over all the sequenced libraries, supporting the notion that the miRNAs are composing the largest population of non-coding RNAs found in the studied human leukocyte samples. Concurrently, we employed splice junction and exon microarrays to generate parallel transcriptome datasets from the same cells in order to identify AS changes in the modified miRNAs predicted targets, as well as the cellular composition changes in patients and post-treatment. Testing the same patients before and after surgery further overcame individual variability. Further studies which will expand the size of the tested cohort will enable validation of our findings in larger and independent patient and healthy volunteer cohorts.

We identified significant changes in 16 miRNAs in PD and in 11 post-DBS treatment, 5 of which were among these changed in the disease, but inversely, reinforcing the disease and treatment relevance of these changes. The post-DBS change was further dependent on the stimulus itself, as 1-h off-stimulus completely changed the modified miRNA profiles as compared with the stimulated state. We also identified expression changes in two newly proposed miRNAs that were recently reported as tRNA fragments, possibly originated from the antisense copy of an endogenous retroviral element (Kawaji et al., 2008; De Hoon et al., 2010). The observed miRNA changes post DBS were compatible with miRNA expression being subject to rapid changes under modified environmental conditions (Brzuzan et al., 2012) and immunological challenges (Perry et al., 2008) and indicating possible relevance to the DBS-suppressed symptoms. To our knowledge, our study is the first to construct differential disease- and treatment-related networks in human cells from neurodegenerative disease patients.

Analysis of gene isoform differential expression, AS and modifications of miRNA binding sites revealed significant differences in splice isoform profiles between PD patients and healthy control volunteers blood leukocytes, as well as in post- compared to pre-DBS states, following the brain stimulation treatment. We identified enrichment of immune cellular composition by lineage cell profiling analysis of the exon microarrays, both pre- and post-DBS. Through database and network analyses we identified disease and treatment networks of miRNAs and spliced targets. Combining the data from differential expression of miRNAs with the changes in expression of their potential targets in a systematic way is a powerful tool to identify disease related miRNA-target gene networks. In this study we combined miRNA expression profile in PD patients pre- and post-treatment with data of splice junction microarrays from the same samples presenting a systems biology approach for studying blood leukocyte RNA expression patterns in neurological and neurodegenerative diseases.

Intriguingly, there were more predicted binding sites in the 3′ UTR as compared with the other regions, compatible with other's observations for more efficient regulation of the target genes through 3′-UTR binding. Whereas binding to the 3′ UTR induces stronger degradation of the target, auxiliary binding to the coding sequence may possibly increase this effect. Recent reports that target regulation through miRNAs may function also in coding regions, not only the 3′ UTR (Dolken et al., 2010; Hafner et al., 2010) are compatible with our predictions. Correspondingly, computational analyses of data produced from RNA-binding protein PAR-CLIP [Photoactivatable-Ribonucleoside-Enhanced Crosslinking and Immunoprecipitation, (Ule et al., 2003)] experiments showed that nearly 50% of the miRNA binding sites were located in the coding regions of the targets, compatible with the capacity of miRNAs to each regulate thousands of target mRNAs (Lewis et al., 2005; Friedman et al., 2009). Yet, the molecular mechanisms underlying the selection of interaction between distinct “seed” domains and single transcripts was not yet interrogated in a global manner in human diseases; therefore, the identification of more than a single miRNA-target putative interaction site opens yet more questions, for future studies.

The state specific enrichment for GO processes suggests disease- and treatment-specific functional involvement. The disease induced processes included ubiquitin ligase complex, mitochondrion organization and regulation of translation, compatible with our recent observations in brains of PD model mice (Soreq et al., 2012a). The DBS induced functional changes included MAPK phosphatase export from nucleus as well as in ubiquitin homeostasis, suggesting treatment influence on disease leukocyte pathways.

While the majority of small RNAs are found within the cells, a growing number of miRNAs are observed outside cells, including various body fluids and blood (Caby et al., 2005; Garcia et al., 2008). For example, small RNA deep sequencing identified miRNAs in exosomes, membrane-bound nanoparticles which are a newly described pathway of intercellular communications (Zhang and Grizzle, 2011), with a distinct signature during prion infection (Bellingham et al., 2012). Circulating miRNAs were recently suggested as new biomarkers in diagnosis, prognosis, and treatment of cancer (Allegra et al., 2012). Moreover, extracellular RNAs have immune-modulating properties (Altincicek et al., 2008) and can escape the blood-brain barrier to reach distal locations (Graner et al., 2009) or to tumors. This suggests a future venue to pursue.

Epigenetic chromatin changes were recently found in blood leukocytes of schizophrenia patients (Melas et al., 2012), where association was found between DNA methylation and antipsychotic drug treatment. As PD also entails hereditary component in about 10% of the cases, it will be highly interesting to pursue in the future for genome-wide DNA methylation changes in PD patients prior to, and following DBS, which may reveal dysregulated epigenome in PD and may further discover DNA methylation disease biomarkers in blood leukocytes.

Concurrent measurements of miRNAs and their mRNA targets were recently reported for two other human diseases: Myelodysplastic Syndromes (Beck et al., 2011) and multiple sclerosis (Angerstein et al., 2012) but these studies did not address splice isoform and AS changes of the targets. To our knowledge, this is the first report where quantitative information about miRNAs has been generated concurrently with particular splice variants in a genome wide scale for blood leukocytes of human patients prior to, and post brain stimulation treatment. Thus, our study provides a useful data set supplementing previous microarray datasets of transcripts, which lacked isoform-specific resolution, and provides a comparative resource for miRNA study in PD.

The PD network included the transcription factor *FOXP1*, implicated in a miRNA-mediated feedback loop controlling the survival of midbrain dopaminergic neurons (Kim et al., 2007), and shown to co-act with *PITX3* for maintaining neuronal fate in the midbrain. Specifically, *PITX3* promotes midbrain identity through regulation by this dopaminergic regulator (De Mena et al., 2010; Konstantoulas et al., 2010). These findings suggest relevance of the observed changes for both the PD phenotype and treatment efficacy, and foresee the use of similar network approaches for the study of other neurological and neurodegenerative diseases.

The identified miRNA changes in PD patient's blood leukocytes here may provide an important biomarker profile, reflecting remarkable dynamics and stimulus-dependent reversal of miRNA expression. Our data set provide a source for miRNA expression in blood leukocytes in healthy state and under neurodegenerative disease. Our analyses and findings suggest disease-associated dys-regulation and treatment-related reversal of the transcriptional mediated checks and balances that, under healthy state functioning, reflect balanced splicing events controlling leukocyte transcript profiles.

## Author contributions

Conceived and designed the experiments: Lilach Soreq, Hermona Soreq, Hagai Bergman, Zvi Israel. Recruited study participants: Zvi Israel, Hagai Bergman, Lilach Soreq. Collected patient samples: Lilach Soreq. Produced RNA: Lilach Soreq. Conducted computational and Bioinformatic analyses: Lilach Soreq. Designed the software used in analysis: Nathan Salomonis, Lilach Soreq. Contributed analysis tools: Nathan Salomonis. Wrote the paper: Lilach Soreq and Hermona Soreq and all co-authors approved.

### Conflict of interest statement

The authors declare that the research was conducted in the absence of any commercial or financial relationships that could be construed as a potential conflict of interest.

## Supplementary material

Figure S1 **The percentage of annotated reads and of the miRBase aligned reads. (A)** Percent of annotated reads of the total number of RNA-Seq reads in all the 12 RNA-Seq libraries. **(B)** Percent of miRBase detected entries in the RNA-Seq libraries **(C)** Percent of sequence match between the reads and the miRBase sequences (perfect, 1 mismatch, 2 mismatches and 3 mismatches) **(D)** Percent of reads match to the human reference sequences database (which comprises of non protein-coding RNAs other than miRNAs, such as SINEs and ALU sequences).

Figure S2 **Expression map of selected miRNAs and miRNA^*^ (3′/5′ forms) in blood leukocytes of Parkinson's patients pre- and post- treatment and of healthy control volunteers.** Read counts of selected miRNAs that were detected through RNA-Seq alignment of small RNAs detected in blood leukocytes of PD patients pre- and post-DBS while being on and off electrical stimulation and of matched healthy control (HC) volunteers to the miRBase database. **(A)** Highly expressed miRNAs **(B)** Highest expressed, and **(C)** Count levels of miRNA^*^ (3^′^/5^′^) forms presenting low expression **(D)** and high expression **(E)**. Y axis: the number of reads. Dark blue: the expression of miRNAs in HC volunteers, lighter blue: in PD patients pre-DBS, light blue: post-DBS while being on electrical stimulation (DBS) and lightest: in PD patients post-DBS following one hour of a complete electrical stimulation cessation (DBS-off).

Figure S3 **DBS differentially expressed miRNAs.** Presented are miRNAs that were detected as changed in PD patients post-DBS as compared with HC volunteers (left), the same patients while being off electrical stimulation state as compared with healthy control volunteers (middle) and in off electrical stimulation state as compared with the pre-surgical state (right). Y axis: fold change multiplied by the ln (log base 10) of the significance p-value which was obtained by a negative binomial distribution test through a generalization of the Poisson model that recaptures biological variance correctly (Robinson and Smyth, 2008).

Figure S4 **Classification of patients pre-DBS from healthy control volunteers.** The junction microarrays expression levels of blood leukocyte RNA exons that were detected as changed in PD patients as compared with HC volunteers correctly classified patient samples apart from the healthy samples. The unsupervised hierarchical classification was conducted on the expression signals of the exons detected by splicing-index analysis which was conducted by a modified version of AltAnalyze (Salomonis et al., 2009) that was adopted to the human splice junction (HJAY) arrays and is based on the measurement described for exon arrays under (Gardina et al., 2006). The classification was conducted with Euclidean distance measure on both the exons and the samples.

Figure S5 **Classification of patients post-DBS from pre-DBS state.** The junction microarrays expression levels of blood leukocyte RNA exons that were detected as changed by splicing-index analysis in PD patients pre-DBS as compared with post-DBS (DBS) state (while being on electrical stimulation) correctly classified between the states. The classification was conducted by a hierarchical classifier with Euclidean distance measure on both the exons and the samples.
