# Supplementary material for: Small RNA sequencing-microarray analyses in Parkinson leukocytes reveal deep brain stimulation-induced splicing changes that classify brain region transcriptomes
Source: Front Mol Neurosci. 2013 May 13;6:10. doi: 10.3389/fnmol.2013.00010 (PMC3652308; doi:10.3389/fnmol.2013.00010)
Supplement: Figure S1 — The percentage of annotated reads and of the miRBase aligned reads. (A) Percent of annotated reads of the total number of RNA-Seq reads in all the 12 RNA-Seq libraries. (B) Percent of miRBase detected entries in the RNA-Seq libraries (C) Percent of sequence match between the reads and the miRBase sequences (perfect, 1 mismatch, 2 mismatches and 3 mismatches) (D) Percent of reads match to the human reference sequences database (which comprises of non protein-coding RNAs other than miRNAs, such as SINEs and ALU sequences). [file Presentation1.PDF]

Small RNA sequencing-microarray analyses in Parkinson leukocytes reveal deep brain stimulation-induced and splicing changes that classify brain region transcriptomes

- Lilach Soreq[1], Nathan Salomonis[2], Michal Bronstein[3], David S. Greenberg[4], Zvi Israel[5], Hagai Bergman[1] and Hermona Soreq[4*] [1]Department of Medical Neurobiology, The Hebrew University of Jerusalem, Israel
- [2]Division of Genomic Medicine, Gladstone Institute of Cardiovascular Disease, USA
- [3]The Institute of Life Sciences, The Hebrew University of Jerusalem, Israel
- [4]Department of Biological Chemistry, The Hebrew University of Jerusalem, Israel
- [5]Department of Neurosurgery, Hadassah University Hospital, Israel

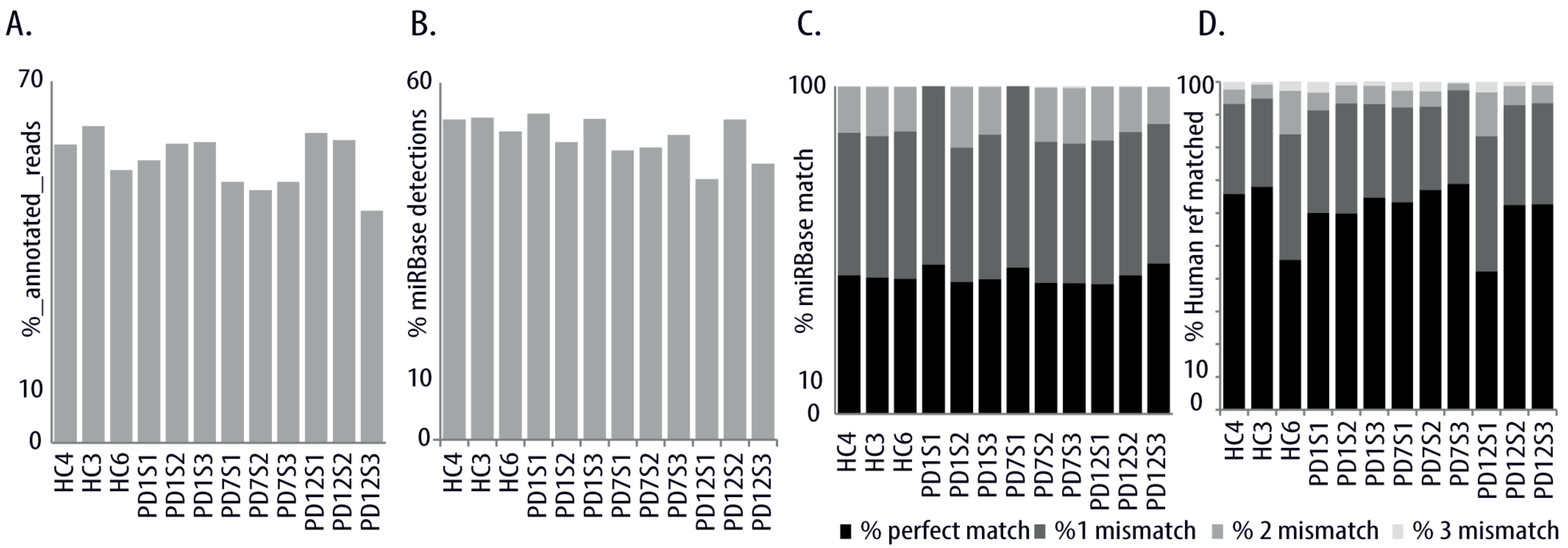
A.
B.
C.
D.
%_annotated_reads
% miRBase detections
% miRBase match
% Human ref matched
70
60
100
10
0
HC4
HC3
HC6
PD1S1
PD1S2
PD1S3
PD7S1
PD7S2
PD7S3
PD12S1
PD12S2
PD12S3
% perfect match
%1 mismatch
% 2 mismatch
% 3 mismatch

Figure S1

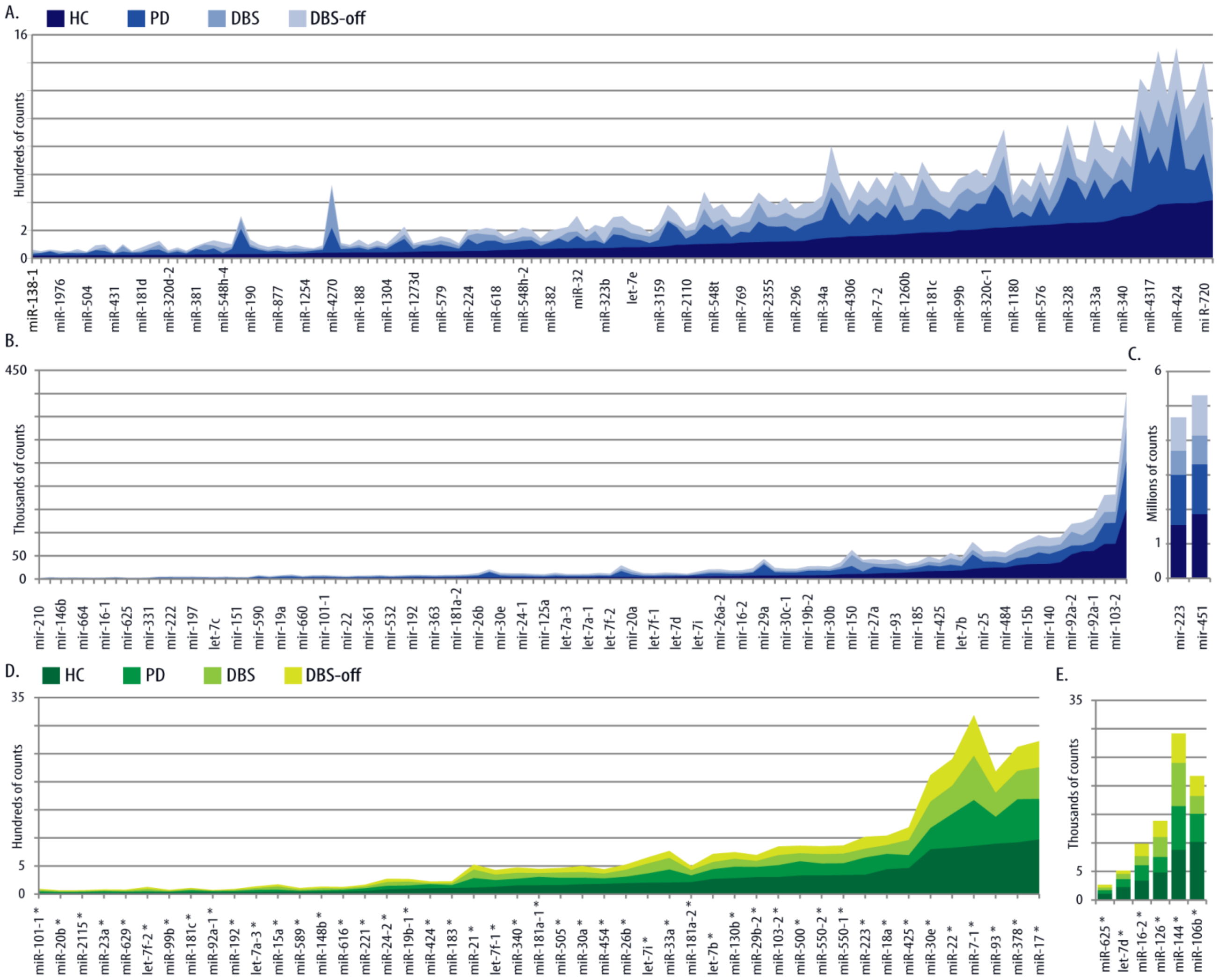

A.
HC
PD
DBS
DBS-off
Hundreds of counts
16
2
0
miR-138-1
miR-1976
miR-504
miR-431
miR-181d
miR-320d-2
miR-381
miR-548h-4
miR-190
miR-877
miR-1254
miR-4270
miR-188
miR-1304
miR-1273d
miR-579
miR-224
miR-618
miR-548h-2
miR-382
miR-32
miR-323b
let-7e
miR-3159
miR-2110
miR-548t
miR-769
miR-2355
miR-296
miR-34a
miR-4306
miR-7-2
miR-1260b
miR-181c
miR-99b
miR-320c-1
miR-1180
miR-576
miR-328
miR-33a
miR-340
miR-4317
miR-424
mi R-720
B.
Thousands of counts
450
50
0
mir-210
mir-146b
mir-664
mir-16-1
mir-625
mir-331
mir-222
mir-197
let-7c
mir-151
mir-590
mir-19a
mir-660
mir-101-1
mir-22
mir-361
mir-532
mir-192
mir-363
mir-181a-2
mir-26b
mir-30e
mir-24-1
mir-125a
let-7a-3
let-7a-1
let-7f-2
mir-20a
let-7f-1
let-7d
let-7i
mir-26a-2
mir-16-2
mir-29a
mir-30c-1
mir-19b-2
mir-30b
mir-150
mir-27a
mir-93
mir-185
mir-425
let-7b
mir-25
mir-484
mir-15b
mir-140
mir-92a-2
mir-92a-1
mir-103-2
C.
Millions of counts
6
1
0
mir-223
mir-451
D.
HC
PD
DBS
DBS-off
Hundreds of counts
35
5
0
miR-101-1 *
miR-20b *
miR-2115 *
miR-23a *
miR-629 *
let-7f-2 *
miR-99b *
miR-181c *
miR-92a-1 *
miR-192 *
let-7a-3 *
miR-15a *
miR-589 *
miR-148b *
miR-616 *
miR-221 *
miR-24-2 *
miR-19b-1 *
miR-424 *
miR-183 *
miR-21 *
let-7f-1 *
miR-340 *
miR-181a-1 *
miR-505 *
miR-30a *
miR-454 *
miR-26b *
let-7i *
miR-33a *
miR-181a-2 *
let-7b *
miR-130b *
miR-29b-2 *
miR-103-2 *
miR-500 *
miR-550-2 *
miR-550-1 *
miR-223 *
miR-18a *
miR-425 *
miR-30e *
miR-22 *
miR-7-1 *
miR-93 *
miR-378 *
miR-17 *
E.
Thousands of counts
35
5
0
miR-625 *
let-7d *
miR-16-2 *
miR-126 *
miR-144 *
miR-106b *

Figure S2

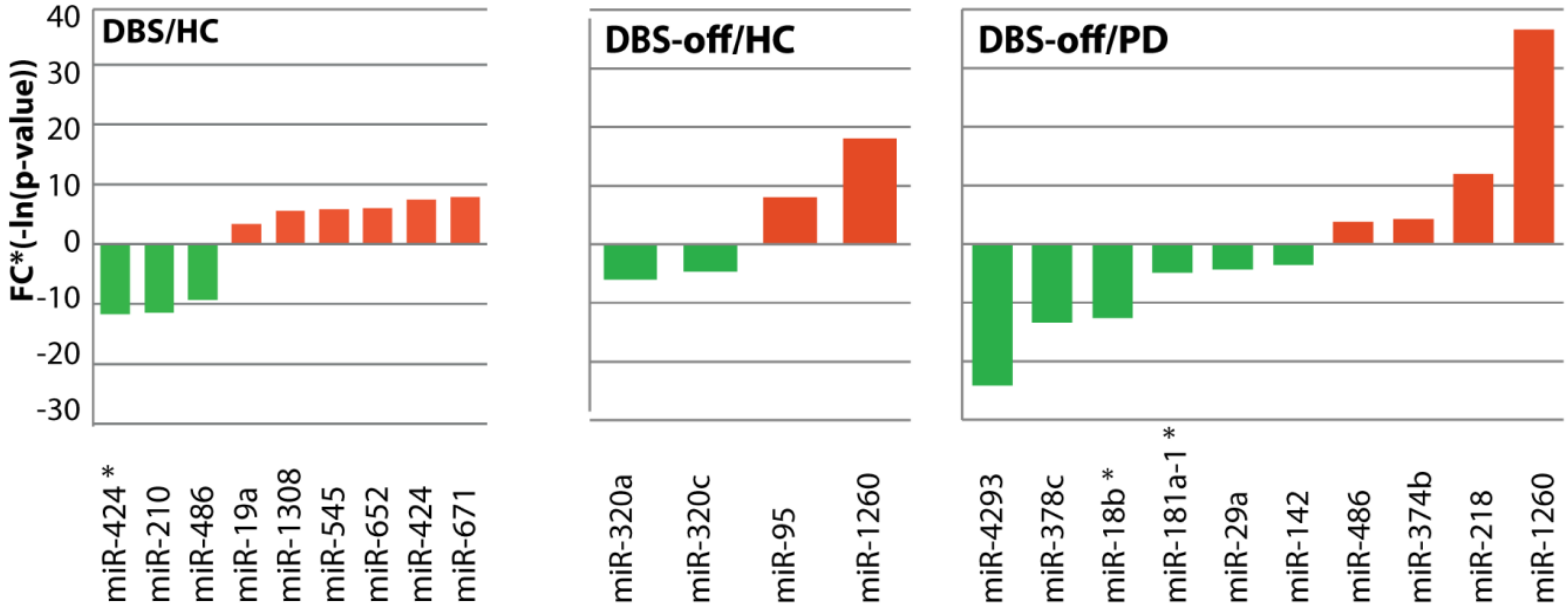

FC*(-ln(p-value))
40
30
20
10
0
-10
-20
-30
DBS/HC
miR-424 *
miR-210
miR-486
miR-19a
miR-1308
miR-545
miR-652
miR-424
miR-671
DBS-off/HC
miR-320a
miR-320c
miR-95
miR-1260
DBS-off/PD
miR-4293
miR-378c
miR-18b *
miR-181a-1 *
miR-29a
miR-142
miR-486
miR-374b
miR-218
miR-1260

Figure S3

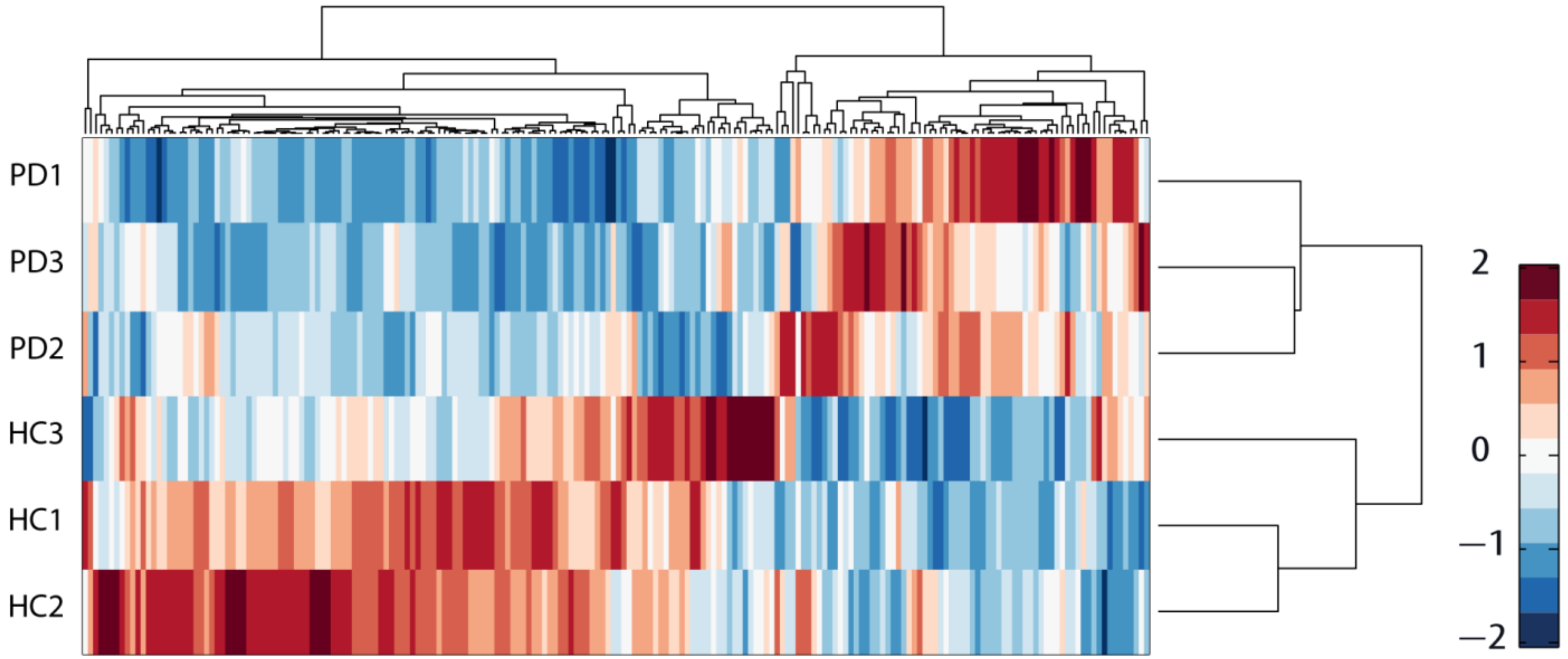

Figure S4

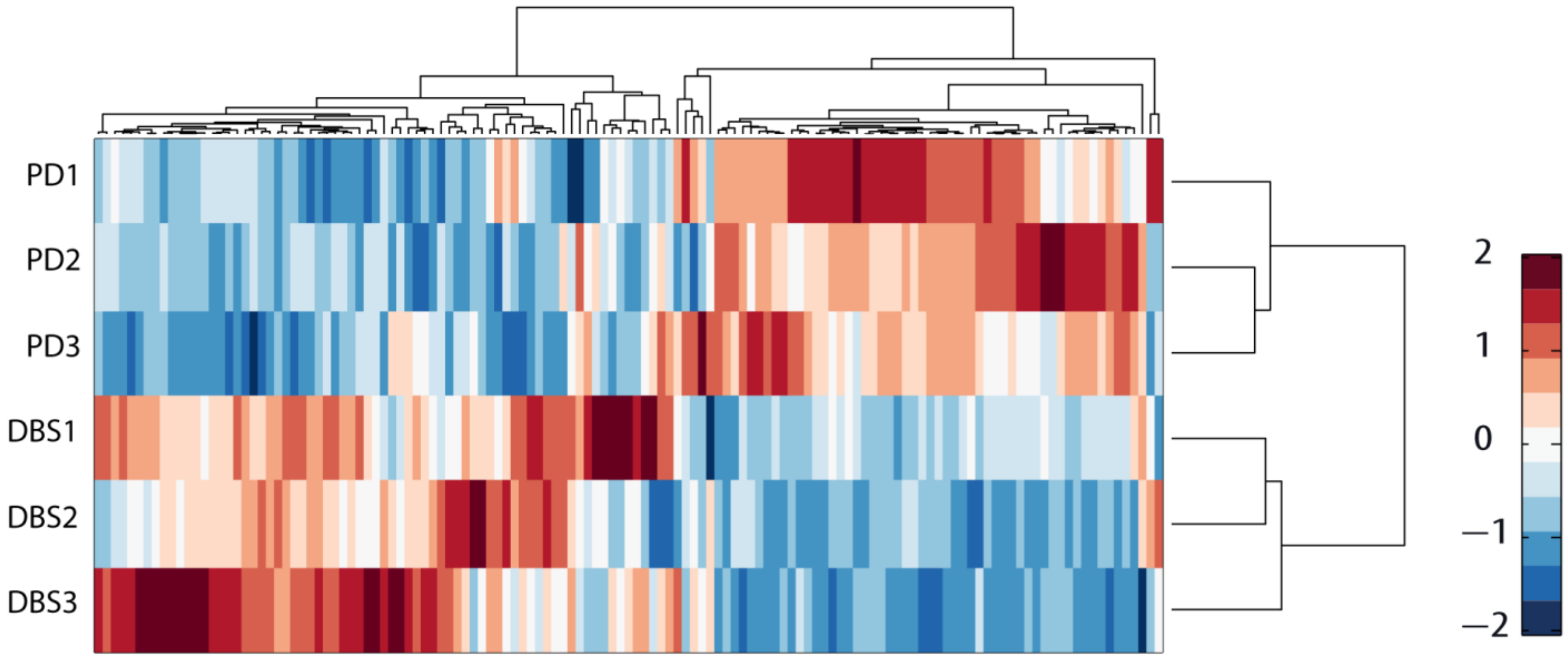

Figure S5
